# Parameter estimation using randomized phases in an integrated assessment model for Antarctic krill

**DOI:** 10.1371/journal.pone.0202545

**Published:** 2018-08-17

**Authors:** Douglas Kinzey, George M. Watters, Christian S. Reiss

**Affiliations:** NOAA Fisheries, Antarctic Ecosystem Research Division, La Jolla, California, United States of America; Texas A&M University, UNITED STATES

## Abstract

An integrated model assessing the status and productivity of Antarctic krill (*Euphausia superba*, hereafter krill) was configured to estimate different subsets of 118 potentially estimable parameters in alternative configurations. We fixed the parameters that were not estimated in any given configuration at pre-specified values. The model was fitted to over forty years of fisheries and survey data for krill in Subarea 48.1, a statistical reporting area around the Antarctic Peninsula used by the Commission for the Conservation of Antarctic Marine Living Resources (CCAMLR). The number of estimated parameters was gradually increased across model configurations. Configurations that estimated more parameters fitted the data better, but the order in which the parameters were estimated became more important in finding the best fit. Twenty-two configurations estimating from 48 to 107 parameters were able to obtain an invertible Hessian matrix that was subsequently used to estimate parameter uncertainty. Parameter uncertainties calculated using asymptotic approximation around the maximum likelihood estimates were often larger than uncertainties based on Markov chain Monte Carlo sampling for the same parameters. Diagnostics applied to MCMC samples in the best model of each configuration that obtained an invertible Hessian indicated that the most highly parameterized configurations did not reach stationary distributions. A 96-parameter configuration was the best fitting model of those that passed the MCMC diagnostics. The ΔAIC and ΔBIC scores indicated essentially no support relative to the best model for the alternative models that also passed MCMC diagnostics. Simulated data using the configurations as operating models showed that while all configurations passed "self-tests" for spawning biomass and recruitment, there was a small negative bias due to model penalties in the fishing mortality estimates for years with the highest fishing mortalities. "Cross-tests" of configurations that estimated different parameters often differed from the operating model values.

## Introduction

Integrated models in fisheries stock assessment are fitted to multiple data sources and may combine different probability models for different types of data in the likelihood function. For example, one type of data might have errors distributed following a lognormal distribution and another type might be distributed as a multinomial. The model parameters are shared between the different likelihood components to minimize the deviance between the model predictions and all the types of data the model is fitted to [1, 2, 3]. These models can be highly parameterized, incorporating individual parameters in multiple nonlinear relationships reflecting the underlying population dynamics. They are used to infer the current status and productivity of a fished stock in comparison to a reference state, and to predict future states of the stock at alternative proposed levels of future harvest. The proposed harvests are removed from the model population described by the parameters as it is projected into the future. These projections can then be compared to management objectives.

The fits of different sets of potential parameter values to the data can be conceptualized as a "likelihood surface", analogous to a three-dimensional topographic map with ridges and valleys. In the case of statistical modeling, instead of three dimensions the likelihood surface has as many dimensions as the model has parameters. The best point on this multidimensional surface (the maximum likelihood) is its highest peak. This represents the combination of parameter values that produce the closest fit to the data. For computational efficiency, the equivalent value of the lowest negative log-likelihood is commonly used to identify the best parameter estimates, so that the lowest point in the negative log-likelihood surface (hereafter the likelihood surface) represents the best fitting parameter values. When this likelihood surface is very uneven, analogous to a rugged topographic map with multiple valleys, numerical optimizers attempting to find the best fitting parameters can sometimes get "stuck" in a local minimum instead of finding the best, global minimum.

In this paper the model equations, likelihood components and pre-specified (fixed) quantities of a model are called its structure. This structure, along with the other parameter values, provides information for each individual parameter estimate. The deviance between the predicted values for the data and the actual data is calculated for each likelihood component using a "loss function", which for example could be sum of squares for Gaussian data, or the sum of the products of bin proportions and the log of bin proportions for multinomial data. The sensitivity of the deviance to changes in individual parameter values allows parameter uncertainty to be measured for a given combination of model and data. A parameter with little impact on model fit as its value is changed has more uncertainty associated with it than a parameter in which small changes produce larger effects on the fit.

Various approaches to statistical inference may be employed in integrated modeling. One is maximum likelihood, in which all the information about the parameters comes from the fit of the model to the data, and "prior" probabilities of parameter values are not specified. A second is Bayesian inference, in which parameters are treated as random variables with prior distributions. Penalized likelihood is a third approach in which penalties in the likelihood on some parameter values are analogous to Bayesian priors, although not all parameters are constrained by priors as would be the case in a fully Bayesian model [4, 5, 6]. Model fitting and evaluation of the model predictions compared to data are central to using such models for inference [7].

The model structure can include some quantities fixed at pre-specified values [8, 9]. As the number of estimated parameters instead of fixed quantities increase, finding unique solutions for them (model convergence) can become increasingly difficult. In this study, models with the same data and likelihood equations but differing combinations of estimated parameters and pre-specified quantities are called "configurations". The pre-specified quantities in a simple configuration may be estimated as parameters in another configuration.

Some parameters, such as natural mortality *M* [10, 11], "steepness" *h* (spawner-recruit productivity) [12, 13], and the standard deviation of recruitment residuals *σ*_*R*_ [14] are commonly pre-specified in fisheries stock assessments. In a compilation of 80 stock assessments conducted by NOAA between 2005 and 2012 [15], *M* was pre-specified in 60 assessments, estimated in 8 assessments, no *M* value was used in 9 assessments, and 3 assessments included some configurations that estimated *M* and others that did not. Steepness was fixed in 29 assessments, estimated in 10, not applicable in 30 assessments, and either not used or estimated externally in 11 assessments.

Fixing parameters at assumed values may facilitate finding unique solutions for the remaining parameters but at the potential costs of prediction bias and poor fit to the data if the pre-specified values supplied to the model are wrong. Fixing parameters to pre-specified values can also predetermine key management reference points, e.g., pre-specifying a value for steepness affects the values of biomass, rate of fishing mortality, and spawners-per-recruit predicted to produce maximum sustainable yield [16, 9].

A second technique for facilitating model convergence in integrated modeling is to use narrow priors, penalty functions, or possibly absolute bounds on the parameter estimates to reduce the range of parameter combinations over which the minimizing algorithm searches [17, 9]. A potential drawback to this method is that these prior assumptions might not include the values that would best fit the data, especially if the priors or bounds have been chosen to facilitate convergence rather than representing pre-existing information about the possible ranges for these parameters.

When the likelihood surface of a model with nonlinear components displays multiple local minima, estimating the parameters in phases can facilitate convergence [18]. A phased approach involves initially estimating only a few parameters in the earliest iterations of the numerical optimizer, while holding the others at pre-specified values, then estimating the remaining parameters in later phases. Once activated, the parameters continue to be re-estimated in each subsequent phase.

Various suggestions for the order in which parameters are estimated in fisheries stock assessment have been proposed. Estimating the parameters that scale the biomass first, followed by other parameters such as those for selectivity, for which reasonable guesses can be pre-specified during the early phases, has been recommended [2]. Similarly, it has been suggested that parameters should be estimated in phases once nonlinear models exceed about 20 parameters, with the least certain parameters estimated early, and then better known parameters estimated in subsequent phases [18]. Estimating parameter values in phases is commonly employed in fisheries assessment models such as Stock Synthesis, an assessment framework used on the U.S. west coast and elsewhere [19, 3]. CASAL, an alternative integrated modeling framework used to assess New Zealand and Antarctic fisheries, also allows the use of phases in parameter estimation [20].

Here we aim to identify trade-offs between supplying pre-specified quantities to an integrated assessment versus estimating them in the assessment model, and to explore the parameter estimation performance associated with these trade-offs. We use a length-based, age-structured population assessment model for Antarctic krill to explore the effects of estimating increasing numbers of parameters. We evaluate the performance using a variety of Bayesian and maximum likelihood metrics, including the fits of the configurations to the data (the negative log-likelihoods), the proportion of replicates estimating the same parameters but in different sequential orders that found the best fitting parameter values, information-theoretic measures (ΔAIC and ΔBIC), and Markov chain Monte Carlo (MCMC) diagnostics of convergence. The overall question is, what combination of potential parameters are estimable with the data available for krill that provide the best fit?

## Materials and methods

The krill model uses penalized likelihood. The model was developed in AD Model Builder (ADMB) [18] and fitted to hydroacoustic indices of krill biomass, research-trawl indices of krill biomass, length-compositions of krill in the research-trawl and fishery-observer samples during 1982 to 2016 and 2000 to 2015, respectively, and annual fisheries catches from CCAMLR Subarea 48.1 (the Antarctic Peninsula) from 1976 to 2015 [21]. Annual biomass densities and length-compositions from a 125,019 km^2^ sampling grid were obtained from a combination of the U.S. Antarctic Marine Living Resources Program (U.S. AMLR) research surveys during 1992 to 2016 [22, 23, 24], German research surveys during 1982 to 1989, and a Peruvian research survey during summer 2014, and extrapolated to the surrounding 630,278 km^2^ CCAMLR Subarea 48.1.

### Assessment model

An earlier version of the integrated assessment model for krill was fitted to only research-survey (trawl and hydroacoustic) data [22]. The effects of harvest removals since the 1970s were assumed to be minor at the population level in that model. The population dynamics and likelihood equations used in the model configurations described here (Supplemental 1, "Model equations") are the same as described in the earlier version for the annual length-compositions (from research trawls), and Beverton-Holt recruitment penalties. The penalty on natural mortality in the earlier modeling (Appendix Eq 9 of [22]) was removed from the current configurations.

In the current configurations, annual krill harvests during 1976 to 2016 and observer measurements of length-compositions from the commercial catch from 2000 to 2014 have been added as additional sources of data. Mortality has been separated into natural and fishing mortalities. Model components representing the Baranov catch equation and population effects of the fishery were incorporated to use the new data. The krill model assumes lognormally distributed errors for the annual biomass indices from six surveys and for the fishery catches. Length-compositions from survey trawls and from the fishery are assumed to have multinomially distributed errors.

All survey and fishery selectivities are logistic functions of age in the current model. Model age-compositions from the fishery were converted to lengths available in the data based on the same von-Bertalanffy age-length conversion as for the research trawls (S1 Text).

### Parameter estimation

Different subsets of 118 potentially estimable quantities in the krill assessment were estimated using variable combinations of pre-specified quantities and estimated parameters. All parameters were initialized to the same set of values that were either obtained from the literature or, when pre-existing values for specific parameters could not be found, from early model runs (Table 1). The quantities that were not activated for estimation in a configuration remained at these pre-specified values. From 3 to 12 parameter groups were activated in phases 1 to 7, with the terminal phase ending at phase 9 (a parameter "group" includes indexed parameters represented by each row of Table 1, although not all of these parameters were estimable in the best configurations). The number of potential permutations of parameter groups in different phase sequences was large. For example, for 12 parameter groups estimated in 7 phases the number of permutations is 12!(12–7)! = 5.7e+10. Only a small sample of the potential permutations were evaluated for each configuration.

**Table 1:** Potentially estimable parameters, their pre-specified, initial values, and bounds, if any, on the estimates. The number of parameters estimated is one per survey for parameters subscripted with an *s* and one per year for parameters subscripted with a *y*. Parameters are mathematically described in the Supplemental Information (S1 Text).

Description (number of parameters)	Parameter	Initial value(s)	Bounds
log(unfished mean recruitment) (1)**	ln(*R*_0_)	16.0	
log(mean recruitment) (1)**	$\ln(\bar{R})$	16.0	
log(annual recruitment deviations) (46)**	$\varepsilon_{y}^{R}$	0	-15, 15
log(recruitment standard deviation) (1)**	ln(*σ*_*R*_)	-0.3567	
log(natural mortality) (1)*	ln(*M*)	-0.2232	-5, 5
summer acoustic survey selectivity (2)**	*α*_*s*_,*β*_*s*_	3.3, 0.06	
trawl and winter acoustic survey selectivity (10)**	*α*_*s*_,*β*_*s*_	1.3, 2.8	
fishery selectivity (2)**	*α*_*f*_,*β*_*f*_	1.3, 2.8	
log(average fishing mortality) (1)**	*μ*^*f*^	-2.3026	
log(annual fishing mortality deviations) (41)*	$\varepsilon_{y}^{f}$	0	-12, 8
fishery catch CV (1)**	*σ*_*C*_	0.1	
log(fishery catchability) (1)**	ln(*q*_*f*_)	0	
steepness (1)*	*h*	0.85	0.21, 1
log(survey catchability) (6)**	ln(*q*_*s*_)	0	
von Bertalanffy growth (3)*	ln(*L*_∞_) ln(*k*) ln(*σ*^*v*^)	4.11, -0.799, 1.459	2.5, 5 -10, 5 0.2, 5

* These initial values are from application of the Generalized Yield Model to krill model (values obtained from CCAMLR Secretariat), or recommended by a panel from the Center for Independent Experts that reviewed our integrated krill model in 2016, except the von Bertalanffy variability of length with age (ln(*σ*^*v*^)), which was based on early model runs.

** These initial values are based on fitting the data in early model runs.

Most of the estimated parameters in Table 1 did not have penalties or priors assigned to them. Seven of the parameters were bounded (Table 1) but not all of these were estimated in all models. Bounds on parameter estimates prevent the minimizing algorithm from searching extreme regions of the potential parameter space. This can be problematic if the model estimates a value to be at a bound, as that might indicate the model could find a better fit outside of the bounds. Estimates of bounded parameters should be checked after estimation to ensure they are not at a bound.

Penalties on the annual recruitment deviations from a deterministic Beverton-Holt relationship with spawning biomass (Λ_1_ to Λ_4_) and on fishing mortality (Λ_5_,Λ_6_) are described in the Supplemental Information (S1 Text). All other parameters in Table 1 were freely estimated without penalties or priors, starting from the initial values.

**Fig 1:**
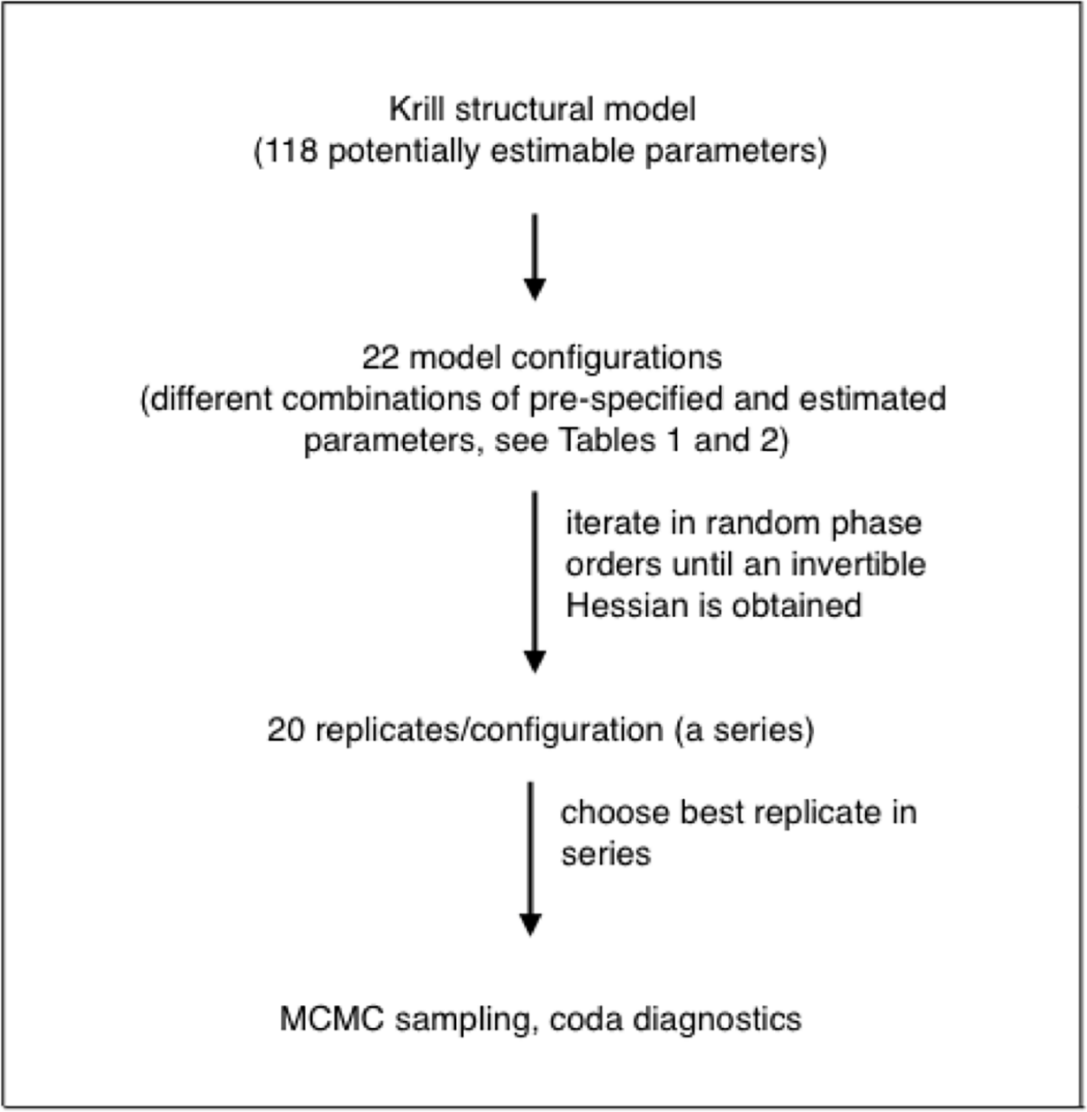
Sequential modeling stages. Relationships among the model, the model configurations, and the phase-randomized replicates.

The active parameters in each configuration were estimated in a series of 20 randomized, sequential phase orderings, or replicates (Fig 1). An R script ran the ADMB executable after randomizing the phases. Each replicate in a configuration estimated the same parameters but in different sequential orders until either an invertible Hessian matrix (an approximation of the covariance matrix for the parameters) was obtained or 3000 iterations of random phase orders were completed. This sequence of randomizing the phase orders in a series of replicates allowed us to evaluate the consistency of the negative log-likelihood values in models that were able to produce MCMC samples (see "Parameter uncertainty: MLE asymptotic approximation and MCMC sampling", below). It also identified phase orderings that produced a local minimum in the negwative log-likelihood instead of the lowest negative log-likelihood observed.

We report results from 22 configurations with from 48 to 107 estimated parameters. Configurations with 108 to 118 estimated parameters were also modeled but these did not produce invertible Hessian matrices. In the first configuration ("cfg") of parameter estimates (cfg I), only recruitment was estimated. The twenty replicates of this configuration each estimated 48 parameters—unfished recruitment, mean recruitment during the estimation period, and 46 annual recruitment deviations (Tables 1 and 2). The other 70 potentially estimable quantities remained at their pre-specified, initial values. The numbers of parameters activated for estimation were increased in subsequent configurations until, for all replicates, the Hessian matrix was no longer invertible (indicating non-convergence) or the maximum gradients were greater than 0.001 (indicating high correlations among some parameter estimates).

**Table 2:** Parameter groupings that were estimated ("e") in each of 22 configurations (“cfg”)*. All configurations were initialized using the parameter values from Table 1. Configurations are ordered by increasing numbers of estimated parameters ("N pars").

cfg	N pars	*R*_0_	$\bar{R}$	$\varepsilon_{y}^{R}$	*μ*^*f*^	*M*	*α*_*f*_,*β*_*f*_	*α*_*s*_,*β*_*s*_	*q*_*f*_	*σ*_*R*_	*h*	*q*_*s*_	*L*_∞_,*k*,*σ*^*v*^
I	48	e	e	e									
II	49	e	e	e		e							
III	49	e	e	e					e				
IV	49	e	e	e						e			
V	49	e	e	e							e		
VI	51	e	e	e		e	e						
VII	54	e	e	e								e	
VIII	63	e	e	e		e	e	e_1-6_					
IX	90	e	e	e	e								
X	91	e	e	e	e	e							
XI	91	e	e	e	e				e				
XII	92	e	e	e	e				e	e			
XIII	93	e	e	e	e	e	e						
XIV	94	e	e	e	e	e	e		e				
XV	96	e	e	e	e	e	e	e_3_	e				
XVI	96	e	e	e	e	e	e	e_3_	e				
XVII	104	e	e	e	e	e	e	e_2-6_	e				
XVIII	104	e	e	e	e		e	e_1-6_			e		
XIX	105	e	e	e	e	e	e	e_1-6_					
XX	106	e	e	e	e	e	e	e_1-6_	e				
XXI	107	e	e	e	e	e	e	e_1-6_	e		e		
XXII	107	e	e	e	e	e	e	e_1-6_	e	e			

* Additional configurations that estimated survey catchability (*q*_*s*_) or growth (*L*_∞_,*k*,*σ*^*v*^) parameters were modeled, but did not have an invertible Hessian matrix and a maximum gradient < 0.001. Subscripts on the survey selectivitys indicate which selectivities were estimated.

Three configurations used different combinations of pre-specified and estimated survey selectivities in a single model. Cfg XV estimated fishery selectivity and catchability, and survey selectivity for the summer Isaacs-Kidd midwater trawls (IKMT) (survey 3). The selectivities for 4 other trawl surveys pre-specified with the values in Table 1. Cfg XVI estimated the selectivity for the same IKMT trawl survey as cfg XV, but the other surveys in cfg XVI were pre-specified with the selectivities estimated in cfg XVII instead of those listed in Table 1. Summer acoustic surveys in cfgs XVI and XVII had pre-specified selectivities of 1 for all age classes (selectivity parameters in Table 1).

Estimating the parameters of a configuration in different phase orders could result in one of three possible outcomes: 1) all the replicates in a configuration could stop after 3000 randomized phase orders without producing an invertible Hessian matrix or standard deviations for the point estimates; 2) some or all replicates could estimate standard deviations for the estimates but be unable to progress to MCMC sampling because the Hessian matrix was not invertible, or; 3) some or all of the replicates could estimate standard deviations, an invertible Hessian matrix, and achieve MCMC sampling of the posterior distribution.

Four metrics were used to evaluate model performance at the maximum likelihood stage for the configurations that were able to progress to MCMC sampling: 1) the negative log-likelihood, a measure of the fit to the data, with smaller numbers indicating better fit; 2) the maximum gradient, a measure of parameter identifiability, with values less than 0.001 preferred; 3) the percentage of replicate models that produced the lowest observed negative log-likelihood in a configuration, a measure of how often a configuration found the best observed negative log-likelihood rather than a local minimum, with higher percentages preferred; and 4) the mean number of phase-randomizations required per replicate before achieving an invertible Hessian matrix and thus MCMC sampling.

The replicate models that produced invertible Hessian matrices within each configuration and among different phase orders were compared. The replicate with the smallest negative log-likelihood and smallest maximum gradient was selected as the best model for that parameter configuration. The best models from each configuration were initially iterated for 10 million MCMC samples, saving every 2,000th sample (thinning), for 5,000 saved samples.

We applied diagnostics from the coda R package [25] to the MCMC samples of the best replicate model from each configuration. These were evaluated using Heidelberger and Welch diagnostics for to up to 27 parameters (when they were estimated). These were all the scalar parameters, the 2015 estimates of spawning biomass and recruitment, and the 2011 estimate for annual fishing mortality deviation. Trace plots, Gweke plots, autocorrelation plots, and density plots indicated whether or not the MCMC sampling appeared sufficient to obtain the stationary distribution for the parameter estimates. MCMC sampling for configurations that failed the convergence tests at 10 million samples were increased to 40 million, and finally to 100 million MCMC samples, and reevaluated using various burn-in (deletion of early samples) and thinning values.

### Parameter uncertainty: MLE asymptotic approximation and MCMC sampling

Identifying the uncertainty associated with the estimates of a statistical model is of fundamental importance in their use [26, 27]. Two methods of quantifying uncertainties in the parameter estimates and derived quantities were employed in this study. The first used the relationship that the inverse of the Hessian matrix for the parameter estimates approximates the covariance matrix for the parameters. An asymptotic, linear approximation of the standard deviations around the parameter estimates can be calculated for nonlinear models from the Hessian matrix using a Taylor series expansion, sometimes called the "delta-approximation method" [28, 7, 6, 29].

MCMC sampling, a second alternative for estimating uncertainty, is a Bayesian method that does not require the assumption of a symmetric Gaussian error distribution used in the asymptotic approximation. ADMB uses the Metropolis-Hastings algorithm for MCMC sampling. The algorithm is supplied with an approximate multivariate normal distribution calculated from the Hessian matrix [29]. Occasionally, a Hessian matrix that is sufficient for the asymptotic approximation will not support MCMC sampling.

### Model uncertainty

Both the asymptotic approximation and MCMC methods of estimating uncertainty assume the model as given, or "true." To the extent the model is not true (model structural uncertainty), both methods may misrepresent the actual uncertainty around the estimates as a representation of nature. Models can be ranked, however, as "approximating models" in their ability to represent a data set by such information-theoretic measures as AIC [30] or BIC [31]. AIC has been described as a predictive-based viewpoint, while BIC measures goodness-of-fit as developed from an explanatory viewpoint [32].

The relationship between achieving a better fit and estimating more parameters was assessed using ΔAIC and ΔBIC model-selection methods. The ΔAIC score was calculated as the difference in twice the negative log-likelihood plus twice the number of estimated parameters between the best model for a data set and alternative models. The ΔBIC score was similarly calculated as the difference in twice the negative log-likelihood plus the log of sample size times the number of estimated parameters. The ΔBIC penalizes increasing numbers of parameters more than the ΔAIC. Sample size for the ΔBIC was 143, calculated as the sum of the number of years of data available from 6 surveys and the fishery (35 years of survey composition data, 54 years of survey biomass indices, 41 years of fishery catches, 13 years of fishery compositions). Alternative models with ΔAIC scores further away from the best model than about 3 or 4 have very little support relative to the best model [30]. The evidence against models more than 10 ΔBIC scores apart is "very strong" [33].

### Simulation testing

Simulation testing of assessment models using "pseudo-data" has been recommended [34, 35]. In this approach, the predicted values generated by a model, the "operating model", are supplied as data to a second model, the "estimating model". The ability of the estimating model to reproduce the parameters of the operating model is then evaluated. When the operating and estimating models have the same structure this evaluation is known as a "self-test" and can be used to examine potential bias in derived parameters such as the time-series of spawning biomass or fishing mortality. When the model structures are different it is known as a "cross-test" and can be used to evaluate process error due to these structural differences.

In this study, pseudo-data were generated without error from the predicted values of each of the model configurations. Years of data available from each data source, effective sample sizes, and CVs were the same as for the real data. Five replicates of each configuration of estimating models were conducted. All configurations were evaluated using self-tests. Cfgs I, V, X, XI and XVI were also evaluated using cross-tests.

## Results

### Increasing the numbers of estimated parameters

Estimating more parameters usually produced a better fit to the krill survey and fishery data, as expected. Twenty-two alternative configurations of those evaluated with 48 to 107 estimated parameters produced invertible Hessian matrices for all 20 of their replicates and were able to be sampled using MCMC. None of the bounded parameter estimates were near their bounds when they were estimated in these configurations. The negative log-likelihoods for models with increasing numbers of parameters decreased (improved) from 17,247 for cfg I to 11,384 for cfgs XXI and XXII ("-LL", Table 3). All of the configurations that passed coda tests estimated an invertible Hessian matrix after the first randomization. Not all of the replicates in a configuration had the same negative log-likelihood, however. As the number of estimated parameters increased, the proportion of replicates that achieved the lowest observed negative log-likelihood decreased (“% best”, Table 3). When more than 96 parameters were estimated, more phase-randomizations were required to obtain an invertible Hessian matrix ("median iterations", Table 3).

**Table 3:** Model fit, consistency and convergence properties for the 22 configurations in Table 2. The number of estimated parameters (N pars), median number of phase randomizations (iterations) before an invertible Hessian was obtained, percentage of the series of replicates that had the lowest objective value (% best), the lowest negative log-likelihood (-LL), maximum gradient (max g), ΔAIC, ΔBIC, and whether or not the configuration passed (p) the coda diagnostics are reported.

cfg	N pars	median iterations	% best	-LL	max g	ΔAIC	ΔBIC	coda
I	48	1	100	17247	3.51E-05	11616	11474	p
II	49	1	100	14223	2.16E-05	5570	5431	p
III	49	1	100	16528	4.19E-05	10180	10041	p
IV	49	1	100	17070	3.02E-05	11264	11125	p
V	49	1	100	17106	2.50E-05	11336	11197	p
VI	51	1	80	13604	1.06E-06	4336	4203	p
VII	54	1	100	16368	3.14E-05	9870	9746	p
VIII	63	13	5	13446	3.67E-03	4044	3946	
IX	90	1	30	13692	3.42E-05	4590	4572	p
X	91	1	95	12054	7.05E-06	1316	1301	p
XI	91	1	100	11766	1.91E-05	740	725	p
XII	92	1	95	11766	2.72E-05	742	730	
XIII	93	1	95	11643	9.36E-06	498	489	p
XIV	94	1	55	11487	1.52E-05	188	182	p
XV	96	1	15	11417	6.66e-05	52	52	p
XVI	96	1	27	11391	3.77E-05	0	0	p
XVII	104	3	30	11384	4.52E-05	2	26	
XVIII	104	19	40	12342	4.10E-05	1918	1942	
XIX	105	8	10	11540	7.06E-02	316	343	
XX	106	2	25	11386	5.84E-05	10	40	
XXI	107	2	35	11383	2.25E-05	6	39	
XXII	107	2	15	11383	1.92E-05	6	39	

**Fig 2:**
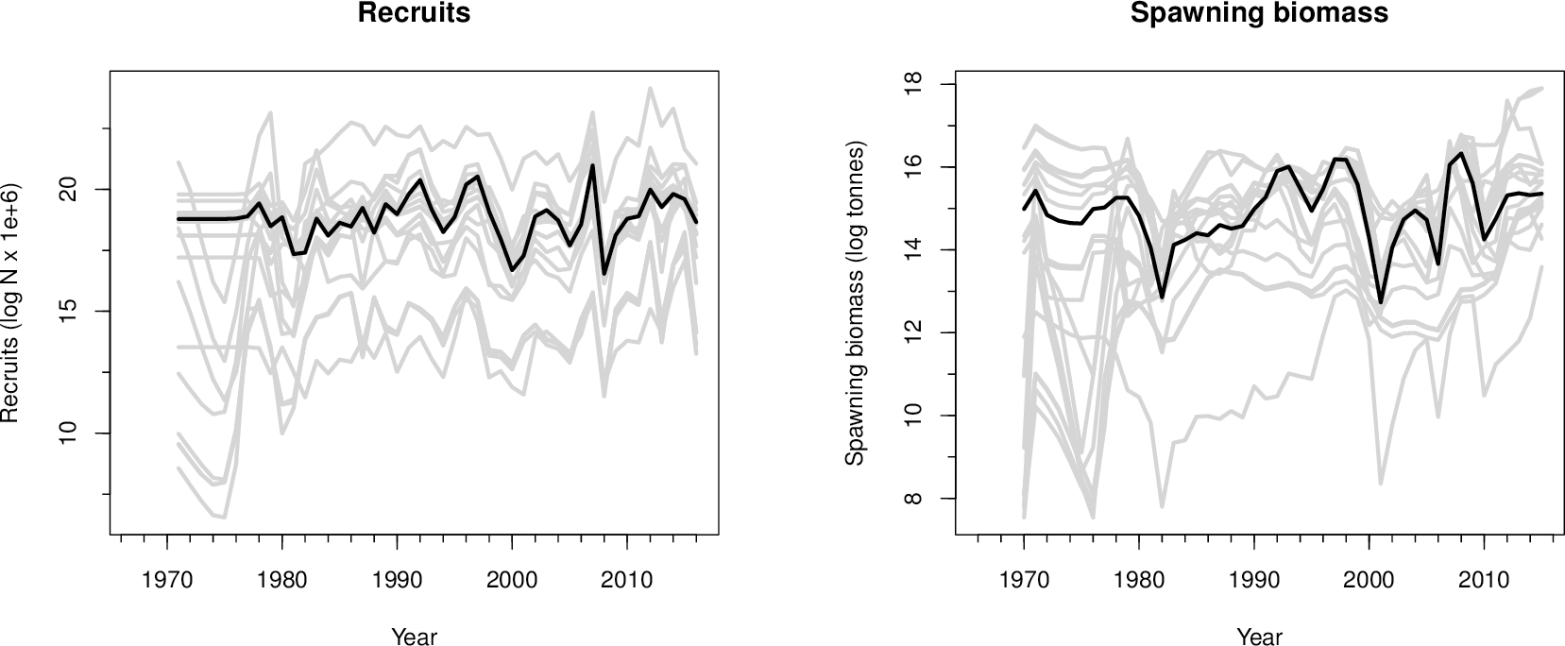
Spawning biomass and recruit numbers from 22 model configurations. Maximum likelihood estimates of spawning biomass and annual recruitment for all 22 configurations (gray), and the best-fitting configuration of those that passed coda diagnostics, cfg XVI (black).

The 22 configurations produced a wide range of spawning biomass and annual recruitment estimates (Fig 2). However, the differences of thousands of negative log-likelihood values among configurations, producing differences in ΔAIC and ΔBIC scores of over 10,000 between the best and worst models, indicate essentially zero support for the fits of the simplest of these configurations compared to the configurations estimating more parameters ("ΔAIC", "ΔBIC", Table 3).

For the configurations estimating fewer than 51 parameters, randomizing the phase order had no effect on model performance. These configurations passed all diagnostic tests with 10 million MCMC samples (Table 3). All 20 replicates in each configuration converged on the first phase-randomization to the same negative log-likelihood value for that configuration (Table 3, cfgs I to V). Maximum gradients were less than 0.0001 for the best replicates and less than 0.001 for all 20 replicates in each of these configurations.

For the 17 configurations in which 51 or more parameters were estimated, obtaining an invertible Hessian matrix could require more than a single randomized phase order for a replicate, and not all the replicates with an invertible Hessian matrix produced the lowest observed negative log-likelihood value for that configuration. For cfg XVI, the median number of phase randomizations before achieving an invertible Hessian matrix was 1, but only 45% of these had the lowest observed negative log-likelihood. The number of replicates for cfg XVI was increased from 20 to 100 to confirm the lowest negative log-likelihood (11,391) for this configuration had been obtained. Twenty-seven of the 100 replicates had the lowest negative log-likelihood, matching the best value observed for the initial 20 replicates.

**Fig 3:**
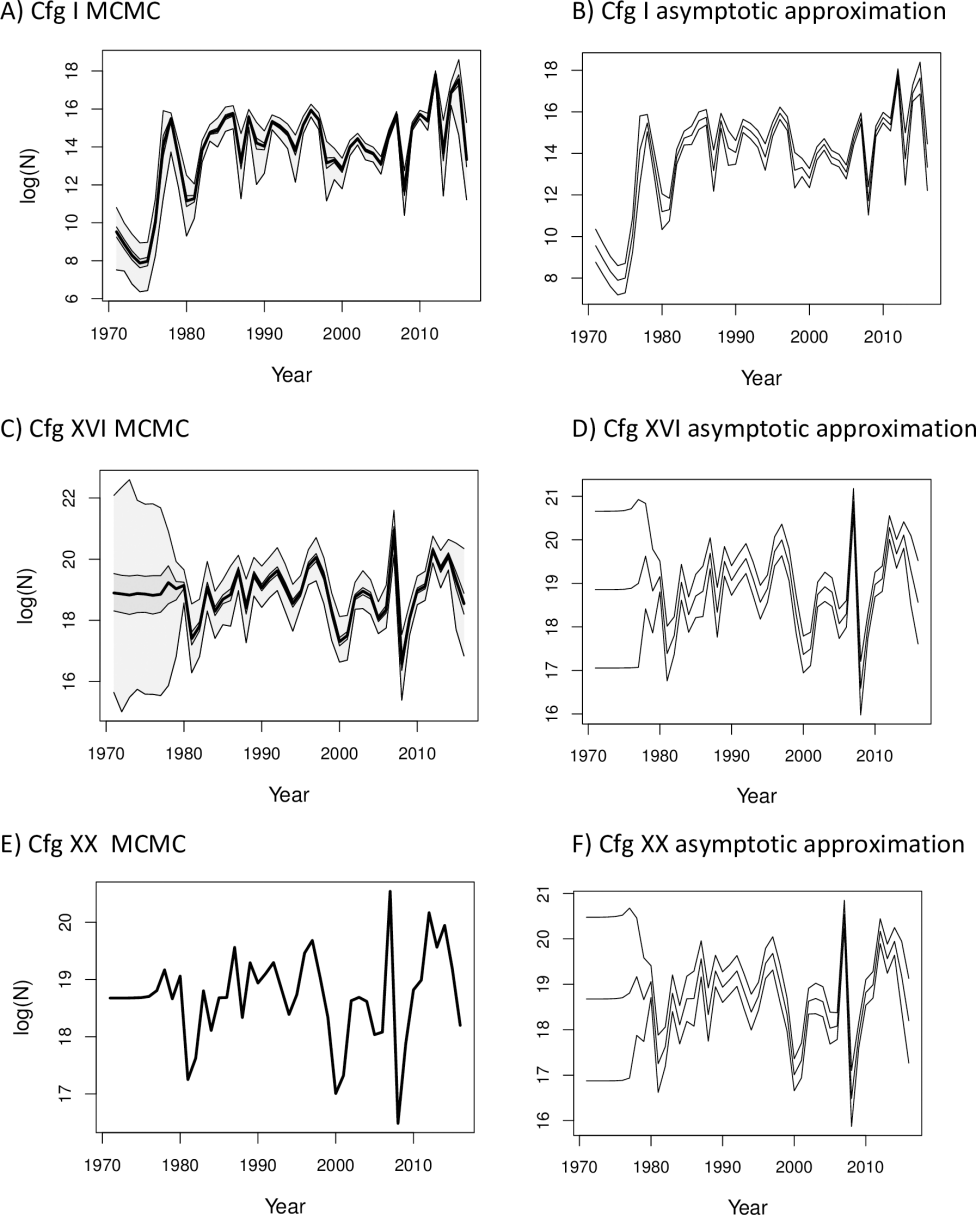
Annual recruitment estimates (where N = number of recruits X 1e+6) and associated uncertainty using MCMC and MLE methods applied to cfgs I, XVI, and XX. MCMC quantiles of 0, 0.25, 0.5, 0.75, and 1.0, and 95% CIs around the MLE from the asymptotic approximation are shown. A) Cfg I MCMC, B) Cfg I asymptotic approximation, C) Cfg XVI MCMC, D) Cfg XVI asymptotic approximation, E) Cfg XX MCMC, F) Cfg XX asymptotic approximation.

**Fig 4:**
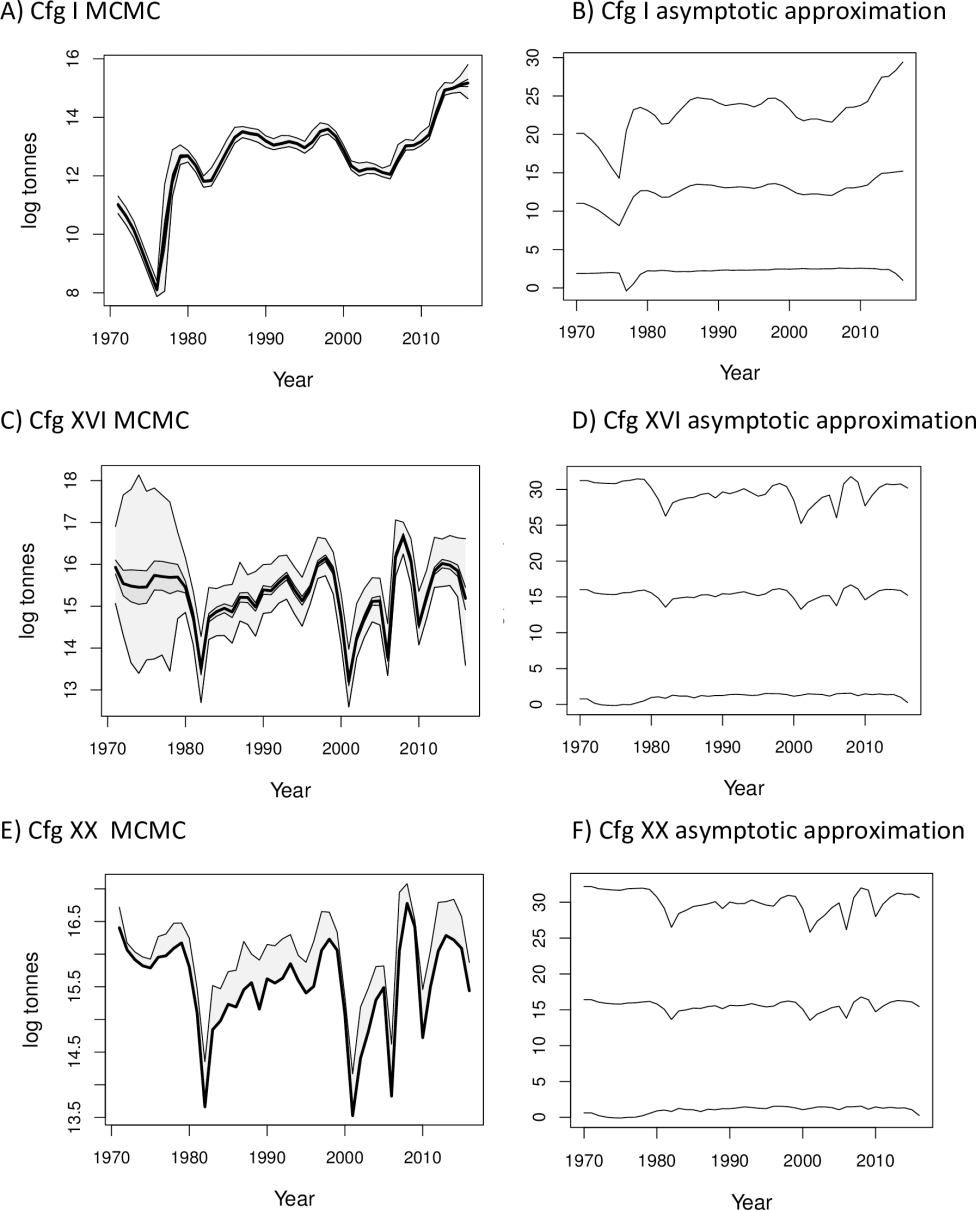
Annual spawning biomass estimates (log tonnes) and associated uncertainty using MCMC and MLE methods applied to cfgs I, XVI, and XX. MCMC quantiles of 0, 0.25, 0.5, 0.75, and 1.0, and 95% CIs around the MLE from the asymptotic approximation are shown. A) Cfg I MCMC, B) Cfg I asymptotic approximation, C) Cfg XVI MCMC, D) Cfg XVI asymptotic approximation, E) Cfg XX MCMC, F) Cfg XX asymptotic approximation.

**Fig 5:**
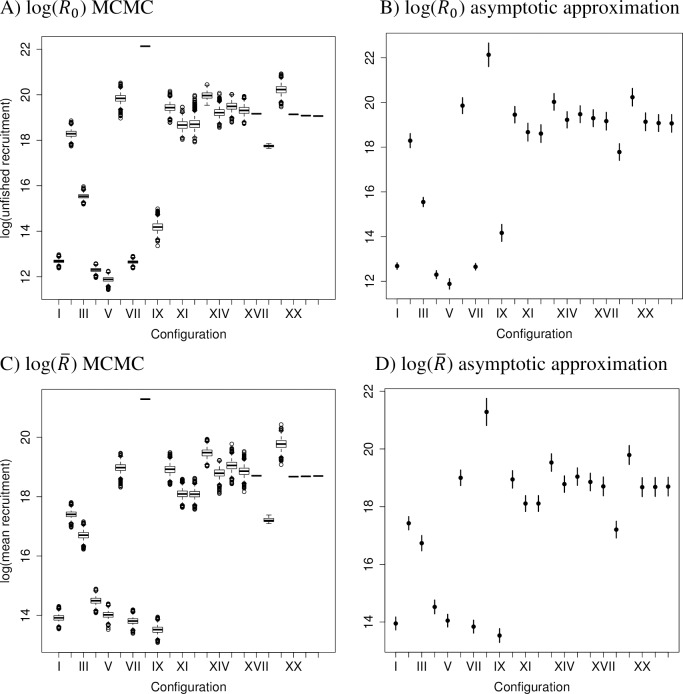
Parameter estimates and uncertainty ranges for unfished recruitment (log(*R*_0_)) and mean log recruitment (log($\bar{R}$)) from 22 model configurations. Boxplots of MCMC distributions, and 95% CIs based on MLE asymptotic approximation around MLE point estimates. A) log(*R*_0_) MCMC, B) log(*R*_0_) asymptotic approximation, C) log($\bar{R}$) MCMC, D) log($\bar{R}$) asymptotic approximation.

**Fig 6:**
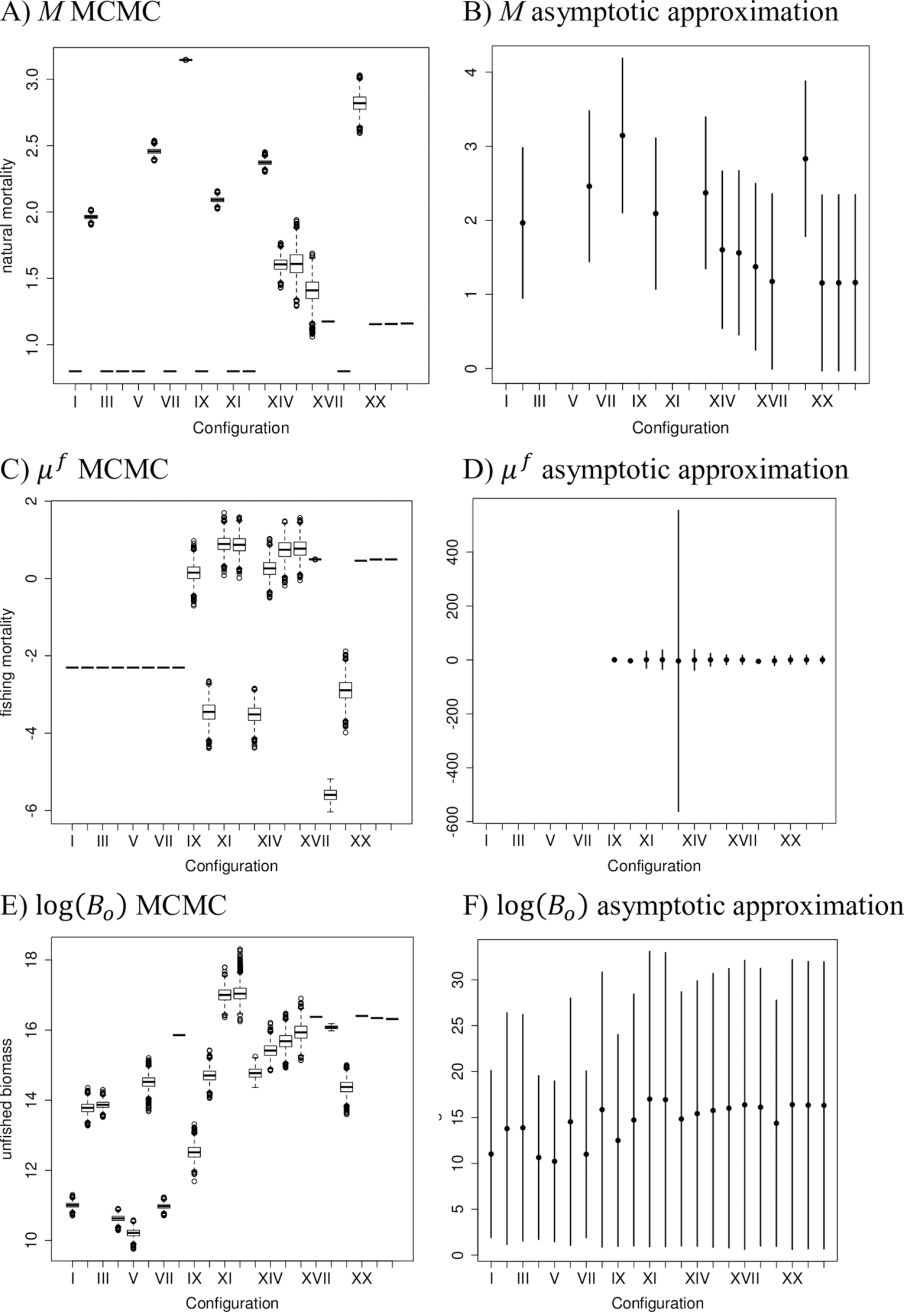
Parameter estimates and uncertainty ranges for natural mortality (*M*), mean fishing mortality (*μ*^*f*^) and unfished biomass (*B*_0_) from 22 model configurations. Boxplots of MCMC distributions, and 95% CIs based on MLE asymptotic approximation around MLE point estimates. A) *M* MCMC, B) *M* asymptotic approximation, C) *μ*^*f*^ MCMC, D) *μ*^*f*^ asymptotic approximation, E) log(*B*_*o*_) MCMC, and F) log(*B*_*o*_) asymptotic approximation.

Cfg XVI, estimating 96 parameters, met the combined criteria of best fitting the data, producing an invertible Hessian matrix, having a maximum gradient less than 0.001, and passing the MCMC diagnostics. None of the configurations that estimated more than 96 parameters passed all coda and other diagnostics, indicating the parameter estimates for those configurations did not converge to a stationary distribution (Table 3). There were substantial differences among the estimates from this best-fitted configuration for the same parameters in many of the other configurations (Figs 2 to 6).

The MCMC samples among cfgs XX, XXI and XXII produced similar estimates of mean annual recruit numbers, natural mortality, average fishing mortality and unfished spawning biomass, and had lower negative log-likelihoods than cfg XVI, but estimated almost no uncertainty in the MCMC estimates for some or most parameters (Figs 3 to 6). Configurations attempting to estimate more than 107 of the 118 total parameters did not produce invertible Hessian matrices. Configurations estimating the parameters for von Bertalanffy growth or for survey catchability had very large maximum gradients or did not produce invertible Hessian matrices when more than one or two other parameters were estimated. The growth and survey catchability parameters were pre-specified at their initial values (Table 1) in all the configurations reported here.

Configurations estimating separate selectivities for all 6 surveys (summer and winter acoustics, summer and winter IKMT trawls, summer and winter RMT8 trawls in cfgs VIII and XVII to XXII) produced invertible Hessian matrices and were able to be sampled using MCMC, but did not pass the Heidelberger and Welch diagnostic test at up to 100 million MCMC samples. In the configurations that attempted to estimate survey selectivities, the summer acoustic selectivities were usually 1 for all ages and the other selectivities were near zero for ages 1 and 2 and increased to 1 by age 4 or 5.

The sequential orders in which parameters were estimated in the replicates for all configurations varied widely without a clear pattern in the phase sequences. In cfg XVI for example, most parameters in the 27 replicates with the lowest negative log-likelihood were first activated in any of phases 1 to 7 (Table 4). The parameter estimates in the best replicates of all the configurations displayed similar variabilities in their phase of estimation.

**Table 4:** Minimum and maximum phases in which parameters were first activated for the 27 best (out of 100 total) replicate randomizations of cfg XVI. All negative log-likelihoods were 11,391, as reported in Table 3 (the other replicates of cfg XVI had negative log-likelihoods from 11,393 to 11,438).

	*M*	*R*_0_	$\bar{R}$	$\varepsilon_{y}^{R}$	*μ*^*f*^	*q*_*f*_	*α*_*s*(3)_,*β*_*s*(3)_	*α*_*f*_,*β*_*f*_
min	2	1	1	1	1	2	1	1
max	7	7	7	7	6	6	7	7

Natural mortality was estimable in many of these configurations but growth was not (the maximum gradients for models including estimated parameters for growth had maximum gradients of greater than 1000). Two parameters traditionally considered "inestimable" within integrated stock assessments, recruitment variability (*σ*_*R*_) and steepness (*h*), were not estimable in this study unless no more than one or two other parameter groups were estimated.

The estimates for natural mortality were higher than the pre-specified value of 0.8 (an annual mortality of 55%, log(*M*) = -0.2232, Table 1), with estimated values of from 1.37 (75% annual mortality—cfg XVI) to 2.46 (91%—cfg VI) in the configurations that estimated natural mortality and passed the coda diagnostics (cfgs II, VI, X, XIII to XVI). The configurations that did not estimate natural mortality fit the data worse than configurations estimating otherwise identical parameter sets, and estimated different trends in spawning biomass during the most recent years modeled than the configurations estimating natural mortality (Tables 2 and 3).

### MCMC and asymptotic variance estimates of uncertainty

MCMC sampling produced more narrow estimates of uncertainty than the variances produced using the asymptotic approximation for many of the estimated parameters. The differences between the MCMC and asymptotic approximation methods of estimating uncertainty were most pronounced for natural mortality (*M*), average log fishing mortality (*μ*^*f*^) and unfished spawning biomass log(*B*_0_) (Fig 6). The derived time series of annual spawning biomass also had much wider credible intervals for the MLE than for the MCMC estimates (Fig 4). For other estimated parameters (e.g., log(*R*_0_), log($\bar{R}$), log($\varepsilon_{y}^{R}$)) the asymptotic and MCMC intervals were similar in location and range.

### Simulation testing

The simulation tests indicated that all configurations were able to reproduce their original estimates of recruitment, spawning biomass, survey and fishery length-compositions, and fishery catches when supplied with pseudo-data in a self-test (e.g., S1 Fig and S2 Fig). In most cases, all five replicates of the estimating models produced the same negative log-likelihood and no cases produced more than 1 replicate with a negative log-likelihood larger than the other 4 replicates. Length-compositions from all six surveys and the fishery fit similarly to the IKMT summer surveys from 1991 to 2002 shown as examples in Figure A in S1 Fig and Figure A in S2 Fig.

When different configurations were used as the operating and estimating models in cross-tests, they often did not reproduce the parameters of the operating model. For example, when cfg XI was the operating model and cfgs I, V, X, XI and XVI were the estimating models, only cfgs X and XI were able to reproduce the recruitment and spawning biomass values of the operating model (S3 Fig). As an estimating model, cfg XVI could closely but not exactly reproduce the parameter values when the other configurations were used as operating models (S3 Fig, S4 Fig). When a simple model such as cfg I was used as an estimating model with a more complex operating model such as cfg XVI the estimates of recruitment, spawning biomass and fishery catches could be far from the operating model values even though the length-composition data were closely fitted (S5 Fig).

There was a small downward bias in the estimates of fishing mortality for the years with the highest fishing mortalities in the operating models. Cfg XVI for example was biased downwards for the highest values of fishing mortality in the self-test (Figures A and B in S6 Fig). This may be due to the penalties on high fishing mortalities being overly severe. Removing them completely allowed the model to fit the most recent years better but caused overestimates in the early years (Figure C in S6 Fig).

## Discussion

With over 40 years of research-survey and fishery data available, the krill population in the vicinity of the Antarctic Peninsula qualifies as a relatively "data-rich" stock. Integrated models based on such a time series could be expected to support the estimation of more parameters than would be possible if time series information about Antarctic krill in the Antarctic Peninsula region was "data-poor" [36, 37]. Estimating differing numbers of parameters for the krill model in different phase orders helped search a larger parameter space and insure that we were able to find the best estimates for as many of the potentially estimable parameters, given the data and model structures, as possible.

The potential for highly parameterized models to exhibit performance issues in fisheries assessment has been previously recognized (e.g., [38, 39]). This study presumes that inference from models that fitted the data better is preferable to inference from models that fitted the data less well, so long as diagnostics do not indicate non-convergence, over-parameterization or similar issues. Careful testing of model estimates using diagnostics such as AIC, BIC, the maximum gradient, coda tests, and simulations are methods of identifying models that may be attempting to estimate more parameters than the data can support.

Despite the wide ranges in parameter estimates from different configurations, differences in ΔAIC or ΔBIC of several thousand or more indicate that there is no support for the configurations, among those evaluated here, that estimated fewer parameters than cfg XVI. In a multi-model inference context, models with ΔAIC or ΔBIC scores more than 10 units away from the best model would be so downweighted when combined with the best model that they would have an essentially undetectable effect on the estimates from the best model when model-averaged [30].

Pre-specified values are structural components of the model, analogous to the choice of a particular function among the various alternatives representing a spawner-recruit relationship, or alternative growth functions. In a statistical model, these choices act as constraints, similar to penalties, on the parameters that were estimated in an individual configuration. Unlike estimated parameters, when these quantities were pre-specified they did not count as parameters for the AIC- or BIC-based model selection criteria.

When more than the 96 parameters in cfg XVI were estimated, declines in model performance and failure to pass coda diagnostics indicated the data were not sufficient to inform the estimates. These configurations appear to be over-parameterized for the available data.

For some derived parameters, e.g., annual spawning biomasses during the time period modeled (*B*_*y*_) (Fig 4), unfished spawning biomass (*B*_0_), natural mortality (*M*) and fishing mortality (*μ*^*f*^) (Fig 6) and the differences between MCMC-based and MLE asymptotic variance-based methods of estimating the uncertainty around the point estimates was striking. The asymptotic approximation produced much wider uncertainty ranges when applied to parameters such as spawning biomass or natural mortality than observed in the MCMC distributions. While other researchers have compared uncertainty estimates for the individual parameters using asymptotic approximations and Bayesian MCMC estimates (e.g., [4, 6]) to our knowledge this is the first study to find such a large discrepancy between the two methods for some parameters. In highly parameterized, nonlinear models, uncertainty estimates based on asymptotic methods may underperform.

The simplest models in this study indicate a potential danger with pre-specifying too many parameters as known quantities in integrated models. When a parameter is pre-specified, it can contribute to the derived and other model estimates without including any uncertainty that would be incorporated if its value had also been estimated. This can produce not only different point estimates than models estimating more parameters, but a falsely narrow estimate of the uncertainty around the model estimates (e.g., compare cfgs I and XVI in Figs 3 and 4). The MCMC distributions for spawning biomass and recruitment in the simpler configurations show different trends than those from the best-fitting and best-performing cfg XVI, and the estimated uncertainties around the parameters from the simple models were misleadingly smaller. If simple models such as cfg I were the only models evaluated the results could encourage unfounded confidence in the estimates.

The simulation tests have several implications. Divergence between the parameter estimates of assessment models using simulated data and the operating model values has been commonly observed in other studies in both self- and cross-tests [34, 35]. In this study, the configurations successfully reproduced the operating model values in self-tests (albeit with a small downward bias in the highest estimates of annual fishing mortalities). The bias in the estimates of the highest annual fishing mortalities (Figure A in S6 Fig) suggests the penalties on the estimates of *F*_*y*_ may need modification.

As expected, the simpler configurations in the simulations were typically unable to reproduce the operating model values from configurations with more parameters because the simple configurations were overly constrained. Even though estimating models with more parameters were able to more closely mimic the simpler operating models than simple estimating models could match complex operating models (S4 Fig, S5 Fig), in general most models failed the cross-tests for recruitment and spawning biomass. Cfg XVI was often close to the operating model values from other configurations (e.g., fitted to the cfg XI operating model, S3 Fig and to the cfg I operating model, S4 Fig).

As an example, operating model cfg I produced recruitments and spawning biomasses that were much lower in the pre-data years than in the years with data. This situation, where the estimates for the pre-data years have very different population dynamics than the years with data is one that the recruitment penalties were designed to drive the model away from. Because cfg I had fewer options for fitting the data than configurations with more parameters, it incurred high recruitment penalties using both the real (S1 Table) and simulated data in a self-test (S2 Table). If the true recruitments were very different in the years immediately prior to the years with composition data, as in an unrecognized regime-shift or similar situation, the recruitment penalties could drive the parameter estimates away from their true values in the pre-data years. In the case of using the cfg XVI estimating model with the cfg I operating model, the cross-test produced the correct trends in the estimates for recruitment and spawning biomass from about 1980 onwards, but increased their magnitude (S4 Fig).

The best model in this paper, cfg XVI, still used 22 pre-specified values for potentially estimable parameters. These were 6 survey catchabilities (*q*_*s*_), 10 survey selectivities (*α*_1,2,4–6_,*β*_1,2,4–6_), steepness (*h*), recruitment variability (*σ*_*R*_), catch variability (*σ*_*C*_) and 3 von Bertalanffy growth parameters (*L*_∞_,*k*,*σ*^*v*^) (Table 1). Because there is no uncertainty associated with these quantities in the model, the uncertainty ranges on the estimated and derived parameters from cfg XVI should be considered minimum values.

Most of the 22 quantities that could not be estimated when many other parameters were also estimated were estimable in simple models with fewer total estimated parameters. These simple models fit the data much more poorly than the configurations reported here, however. One potential option that we did not explore in this study would be to fix parameters that are inestimable in the more highly parameterized configurations to the values estimated in simpler configurations and compare the fits of these models to the fits using the initial pre-specified values from the literature or from early model runs.

## Conclusions

We recommend that researchers and stock assessment scientists who aim to achieve the best fit of integrated models to multiple data sources while minimizing variance should consider using techniques such as phase randomization, ΔAIC and ΔBIC diagnostics, MCMC rather than asymptotic approximations around the MLE estimates, diagnostic tests such as those in the R package coda to ensure convergence of the parameter estimates, and simulation testing. These measures will help guard against applying models that are too simple (e.g. Fig 3A and 3C) or too complex (Fig 5A and 5C) for the available data, and use parameter estimates from models with sufficient complexity to be supported by the data (Fig 4A and 4C).

## Supporting information

S1 Text Model equations. (DOCX)

S1 Fig Cfg I self-test with simulated data. A) Summer IKMT pseudo-data for length compositions from 1991 to 2002 (vertical bars) and fits (lines). B) Recruitment and spawning biomass in the operating model (solid lines) and estimating model (dashed). C) Fits (lines) to fishery catches (points).
(DOCX)

S2 Fig Cfg XVI self-test with simulated data. A) Summer IKMT pseudo-data for length compositions from 1991 to 2002 (vertical bars) and fits (lines). B) Recruitment and spawning biomass in the operating model (solid lines) and estimating model (dashed). C) Fits (lines) to fishery catches (points).
(DOCX)

S3 Fig Cross-tests for recruitment and spawning biomass using simulated data. Cfg XI was the operating model and cfgs I, V, X, XI and XVI were estimating models.
(DOCX)

S4 Fig Cross-test using cfg I as the operating model and cfg XVI as the estimating model. A) Summer IKMT pseudo-data for length compositions from 1991 to 2002 (vertical bars) and fits (lines). B) Recruitment and spawning biomass in the operating model (solid lines) and estimating model (dashed). C) Fits (lines) to fishery catches (points).
(DOCX)

S5 Fig Cross-test using cfg XVI as the operating model and cfg I as the estimating model. A) Summer IKMT pseudo-data for length compositions from 1991 to 2002 (vertical bars) and fits (lines). B) Recruitment and spawning biomass in the operating model (solid lines) and estimating model (dashed). C) Fits (lines) to fishery catches (points).
(DOCX)

S6 Fig Estimating operating model values from cfg XVI of the product of fishery catchability and fishery mortality *ln*(*q*_*f*_)*F*_*y*_ fitted to pseudo-data. (A) self-test (B) self-test with the fishing mortality penalties (Λ_5_,Λ_6_ in S1 "Model equations") removed, and (C) cross-tests with other configurations that estimated fishing mortality.
(DOCX)

S1 Table Likelihood components for the original cfg I and cfg XVI fits to real data. Components are summed over catches, surey indices, compositions from surveys and the fishery, and penalties on F and recruitment.
(DOCX)

S2 Table Likelihood components for simulated data summed over catches, survey indices, compositions from surveys and the fishery, and penalties on F and recruitment for cfg I and XVI. The first Roman numeral in the row names is the operating model and the second is the estimating model (i.e. "cfg_I_XVI" was operating model I and estimating model XVI).
(DOCX)
