# Supplementary material for: Parameter estimation using randomized phases in an integrated assessment model for Antarctic krill
Source: PLoS One. 2018 Aug 17;13(8):e0202545. doi: 10.1371/journal.pone.0202545 (PMC6097675; doi:10.1371/journal.pone.0202545)
Supplement: S1 Text — (DOCX) [file pone.0202545.s001.docx]

**S1 Text. Model Equations**

The number of krill of a given age *a* each year *y* is:

$N_{a,y}=\left\{ \begin{aligned} e^{\bar{R}+\varepsilon_{y}^{R}}\text{ } \text{ }\text{ }\text{ if }a\text{ = 1 }\text{ }\text{ }\text{ }\text{ }\text{ }\text{ } \\ \left( N_{a-1,y-1}e^{-Z_{a-1,y-1}} \right)\text{ } \text{ if 1 < }s< A\text{ }\text{ }\text{ }\text{ }\text{ (1)} \\ \left( N_{A-1,y-1}e^{-Z_{A-1,y-1}}+N_{A,y-1}e^{-Z_{A,y-1}} \right)\text{ }\text{ }\text{if }a = A\text{ } \text{ }\text{ }\text{ }\text{ }\text{ } \end{aligned} \right.$

where $N_{a,y}$ is the number of animals of age *a* at the start of year *y*, $\bar{R}$ is the logarithm of mean recruitment, $\varepsilon_{y}^{R}$is the logarithm of the normally-distributed deviation from the mean recruitment in year *y*, calculated as:

$$\varepsilon_{y}^{R}\sim N\left( \frac{-\left( \sigma_{R} \right)^{2}}{2},\left( \sigma_{R} \right) \right)\text{,}$$

where $\sigma_{R}$ is the pre-specified recruitment variability, $Z_{a,y}$ is the total mortality rate (including permanent emigration and fishing) for animals of age *a* during year *y*, $e^{-Z_{a,y}}$ is the survival rate for animals of age *a* during year *y*, and $A$ is the oldest age considered (the plus group).

***Logistic selectivity***

Survey selectivity *S* by survey *s* for different ages of krill *a* is modeled as a logistic function (a two-parameter form). The proportion of krill at age that are vulnerable to the surveys is calculated as:

$$S_{s,a}=\frac{1}{1+e^{-\beta_{s}\left( a-\alpha_{s} \right)}} \left( 2 \right)$$

where $\beta_{s}\text{ and }\alpha_{s}$ are estimated parameters for survey *s* representing slope and location, respectively. A similar calculation, replacing the survey index *s* with the fishery *f*, is made for the proportion at age that are vulnerable to the fishery.

***Fishing***

Annual fishing mortality during 1976 to 2016 was represented in the calculations of total mortality $Z_{a,y}$ for krill of age *a* in year *y*, along with natural mortality *M*, as:

$$\text{ }Z_{a,y} = F_{a,y}+M \text{(3)}$$

Fishing mortality-at-age $F_{a,y}$ was derived from estimates of the logarithm of average fishing mortality ($\mu^{f})$ and its annual deviation ${(\varepsilon}_{y}^{f})$, mediated by fishery selectivity $S_{a}^{f}$:

$$F_{a,y}=S_{a}^{f}e^{\mu^{f}+\varepsilon_{y}^{f}}$$

The objective function consists of four likelihood components (biomasses from survey indices, length-compositions from surveys, length-compositions from fisheries, and catches) and six penalty functions (four Beverton-Holt recruitment and two fishing mortality penalties).

***First likelihood component: Survey biomass by survey, and year***

${\text{ }L}_{1}=\sum_{y} \sum_{s} \left( \frac{\left( \text{ln}\left( I_{y}^{s} \right)-\text{ln}\left( \hat{I_{y}^{s}} \right) \right)^{2}}{2\left( \sigma^{I^{s}} \right)^{2}} \right)$ (4)

where $\left( \sigma^{I^{s}} \right)^{2}$ is calculated from the CV of the biomass index *I* for survey *s* as $\text{ln}\left( \text{CV}^{2}+1 \right)$, $I_{y}^{s}$ is the total krill biomass calculated from the survey *s* during year *y*, $\hat{I_{y}^{s}}$is the model-estimate of survey biomass during year *y* calculated as $\hat{I_{y}^{s}}=\sum_{a} \left( {S_{s,a}W_{a}N_{a,y}^{s}}/\left( 1+e^{-\text{ln}\left( q_{s} \right)} \right) \right)$, $q_{s}$ is the catchability of survey *s*, initially estimated as $\text{ln}\left( q_{s} \right)$ and constrained to be between 0 and 1 by exponentiating it as a logistic function, $W_{a}$ is weight at age,$N_{a,y}^{s}$ is the number of krill of age *a* during the time of survey *s* calculated similarly to Eq. 1 except that $-Z_{a-1,y-1}$ is replaced with $\left( -Z_{a-1,y-1} \right)^{\rho}$ where $\rho$ is the fraction of the year in which the survey occurred [$\rho$ = (survey month-1)/12, where the first month of the biological year (October) is 1].

***Second likelihood component: length-compositions by survey and year***

$L_{2}=\sum_{y} \left( n_{y}^{s}\sum_{l} \left( L_{l,y}^{s}\text{ln}\left( \hat{L_{l,y}^{s}} \right) \right) \right)$ (5)

where $n_{y}^{s}$ is the effective sample size of survey *s* and year *y*, $L_{l,y}^{s}$is the proportion of krill in length bin *l* during the time of survey *s*, $\hat{L_{l,y}^{s}}$ is the model-estimate of the proportion of krill in length bin *l* during the time of survey *s* calculated as:

$$\hat{L_{l,y}^{s}}=\frac{N_{a,y}^{s}{e^{\left( -Z_{a-1,y-1} \right)}}^{\rho}S_{a}\chi_{a}}{\sum_{h=A_{R}}^{A} {{N_{h,y}^{s}e}^{\left( -Z_{h-1,y-1} \right)}}^{\rho}S_{h}\chi_{h}}$$

$A_{R}$ is the minimum age in the samples, $\chi_{a}$ is the age to length transition for age *a* calculated as:

$$\chi_{a}=\frac{\left[ \Phi\left( \left( \left( l+1 \right)-{L_{\infty}\left( 1-e^{-ka} \right)}/{\sigma^{v}} \right)-\Phi\left( l-{L_{\infty}\left( 1-e^{-ka} \right)}/{\sigma^{v}} \right) \right) \right]}{\sum_{a} \left( L_{\infty}\left( 1-e^{-ka} \right) \right)} (6)$$

where $\Phi$is the cumulative normal distribution, $L_{\infty}, K$ are the von Bertalanffy growth parameters, and $\sigma^{v}$ is the von Bertalanffy standard error.

***Third likelihood component: length-compositions of the fishery***

The fishery length-compositition likelihood $L_{3}$ is calculated using the same equations as described in $L_{2}$for the surveys.

***Fourth likelihood component: catches***

Catch data $C_{y}$, annual catch biomasses reported in the CCAMLR Statistical Bulletin [34] during 1976 to 2016 were compared to model estimates in the catch likelihood:

$$L_{4}=\frac{\sum_{y} \text{ln}\left( {C_{y}}/{\hat{C}_{y}} \right)}{2\sigma_{C}}$$

where the model estimate $\hat{C}_{y}$ was calculated as the sum of the product of annually estimated catch-at-age $\hat{c}_{a,y}$ and weight-at-age $W_{a}$:

$$\hat{C}_{y}=\sum_{a} \left( \hat{c}_{a,y}W_{a} \right)$$

with $\hat{c}_{a,y}$ calculated for the years with data as the product of fishery catchability $q_{f}$ and the Baranov catch equation with numbers-at-age $N_{a,y}$, estimated fishing mortality $F_{a,y}$, and total mortality $Z_{a,y}$:

$$\hat{c}_{a,y}=q_{f}N_{a,y}\frac{F_{a,y}}{Z_{a,y}}\left( 1-e^{{-Z}_{a,y}} \right)$$

Fishery catchability $q_{f}$ in this formulation represents the fraction of the total stock size over all years for which fishing mortality applies. The standard error of the catches $\sigma_{C}$ was pre-specified based on an assumed CV of 0.1. Preliminary model runs indicated that this value produced reasonable fits to most years of catch data as well as the other data sources in the likelihood.

All survey and fishery selectivities are logistic in the current model. Age-compositions from the fishery were converted to lengths based on the same von-Bertalanffy age-length conversion as for the research trawls (Appendix Eqs. 5, 6 in [21]).

***Penalties*** $\boldsymbol{\Lambda}_{\boldsymbol{1-6}}$***: lognormal deviations from Beverton-Holt recruitment, fishing mortality***

The first recruitment penalty is for deviations of mean recruitment during the period of fishing $\bar{R}$ from pre-fishing mean recruitment $R_{0}$:

$$\Lambda_{1}\text{= }0.5\left( R_{0}\text{-}\bar{R} \right)^{2}$$

A second penalty is for deviations from Beverton-Holt recruitment:

$$\Lambda_{2}=0.5\left( \sigma_{R} \right)^{2}\sum_{y} \left( \text{ln}\left( \frac{N_{1,y}}{\hat{R_{y}}} \right)+{0.5\left( \sigma_{R} \right)}^{2} \right)^{2}+Y\text{ln}\left( \sigma_{R} \right)$$

where *y* = model years 1982 to 2016, *Y* = 35 (the number of model years y), $N_{1,y}$ is the model estimate of recruitment (${N_{1,y}=e}^{\bar{R}+\varepsilon_{y}^{R}}$) and $\hat{R_{y}}$ is the predicted Beverton-Holt recruitment at the start of year *y* calculated as:

$$\hat{R_{y}}=\frac{4hR_{0}B_{y}}{\left( 1-h \right)B_{0}+\left( 5h-1 \right)B_{y}}$$

*h* is steepness (the stock productivity at 20% of unfished biomass), $R_{0}$ is unfished recruitment penalized for deviations from mean recruitment as $0.5\left( \text{ln}\left( R_{0} \right)-\text{ln}\left( \bar{R} \right) \right)^{2}$, $B_{y}$ is spawning biomass at the start of year *y*, $B_{0}$ is unfished spawning biomass, and *Y* is the number of recruitment years modeled.

A third penalty is for departures from the pre-specified recruitment deviation $\sigma_{R}$ in the early years before length-composition data were available:

$$\Lambda_{3}=\frac{\sum_{y} \left( \varepsilon_{y}^{R} \right)^{2}}{2\left( \sigma_{R} \right)^{2}}+Y\text{ln}\left( \sigma_{R} \right)$$

where *y* = model years 1971 to 1981, *Y* = 10, $\varepsilon_{y}^{R}$ is the recruitment deviation during year *y*, and $\sigma_{R}$ is the pre-specified recruitment deviation.

A final recruitment penalty was on overall variability in recruitment:

$$\Lambda_{4}=\sum_{y} \left( e^{\varepsilon_{y}^{R}} \right)^{2}$$

where *y* = model years 1971 to 2016.

An earlier penalty constraining the estimates for natural mortality (Eq. 9 in Kinzey et al. 2015) has been dropped from the current model.

***Fishing mortality penalties***

Two penalties, $\Lambda_{5}$ and $\Lambda_{6}$, were applied to limit extreme deviations in the estimates of annual fishing mortality during the years for which it was estimated:

$$\Lambda_{5}=\psi{\sum_{y} \left( e^{\mu^{f}+\varepsilon_{y}^{f}}-0.2 \right)}^{2}$$

where $\psi$ = 10 for phases 1 and 2, and then $\psi$ = 0.001 for all subsequent phases, and

$$\Lambda_{6}=20\left( \bar{\varepsilon}^{f} \right)^{2}$$

where $\bar{\varepsilon}^{f}$ is the mean of the annual deviations in fishing mortality, $\varepsilon_{y}^{f}$.
