# Supplementary material for: Parameter estimation using randomized phases in an integrated assessment model for Antarctic krill
Source: PLoS One. 2018 Aug 17;13(8):e0202545. doi: 10.1371/journal.pone.0202545 (PMC6097675; doi:10.1371/journal.pone.0202545)
Supplement: S1 Fig — A) Summer IKMT pseudo-data for length compositions from 1991 to 2002 (vertical bars) and fits (lines). B) Recruitment and spawning biomass in the operating model (solid lines) and estimating model (dashed). C) Fits (lines) to fishery catches (points). (DOCX) [file pone.0202545.s002.docx]

A)

B)

C)
