# Supplementary material for: Parameter estimation using randomized phases in an integrated assessment model for Antarctic krill
Source: PLoS One. 2018 Aug 17;13(8):e0202545. doi: 10.1371/journal.pone.0202545 (PMC6097675; doi:10.1371/journal.pone.0202545)
Supplement: S6 Fig — (A) self-test (B) self-test with the fishing mortality penalties (Λ5,Λ6 in S1 "Model equations") removed, and (C) cross-tests with other configurations that estimated fishing mortality. (DOCX) [file pone.0202545.s007.docx]

A) B)

C)
