# Supplementary material for: Parameter estimation using randomized phases in an integrated assessment model for Antarctic krill
Source: PLoS One. 2018 Aug 17;13(8):e0202545. doi: 10.1371/journal.pone.0202545 (PMC6097675; doi:10.1371/journal.pone.0202545)
Supplement: S1 Table — Components are summed over catches, surey indices, compositions from surveys and the fishery, and penalties on F and recruitment. (DOCX) [file pone.0202545.s008.docx]

|  | catches | survey biomass | length-compositions | F penalties | recruitment penalties | -LL |
| --- | --- | --- | --- | --- | --- | --- |
| cfg I | 1616.0 | 869.2 | 14412.9 | 0 | 238.7 | 17247.2 |
| cfg_XVI | 6.3 | 50.7 | 11273.8 | 16.7 | 43.1 | 11391.2 |
