# Supplementary material for: Parameter estimation using randomized phases in an integrated assessment model for Antarctic krill
Source: PLoS One. 2018 Aug 17;13(8):e0202545. doi: 10.1371/journal.pone.0202545 (PMC6097675; doi:10.1371/journal.pone.0202545)
Supplement: S2 Table — The first Roman numeral in the row names is the operating model and the second is the estimating model (i.e. "cfg_I_XVI" was operating model I and estimating model XVI). (DOCX) [file pone.0202545.s009.docx]

|  | catches | survey biomass | length-compositions | F penalties | recruitment penalties | -LL |
| --- | --- | --- | --- | --- | --- | --- |
| cfg_I_I | 34.6 | 0.1 | 10207.8 | 0 | 226.1 | 10468.6 |
| cfg_I_XVI | 0 | 2.9 | 10213.6 | 0 | 39.6 | 10256.1 |
| cfg_XVI_I | 674.4 | 2772.9 | 10647.6 | 0 | 57.7 | 14152.5 |
| cfg_XVI_XVI | 0.8 | 0.6 | 10544.5 | 8.2 | 29.4 | 10583.4 |
