# Supplementary material for: Parameter estimation using randomized phases in an integrated assessment model for Antarctic krill
Source: PLoS One. 2018 Aug 17;13(8):e0202545. doi: 10.1371/journal.pone.0202545 (PMC6097675; doi:10.1371/journal.pone.0202545)
Supplement: Supplementary file 6 [file pone.0202545.s004.docx]

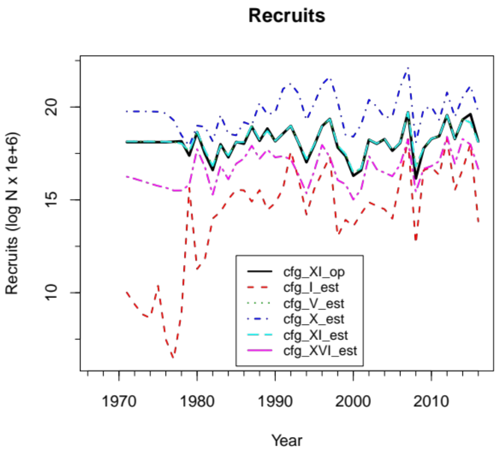
Recruits
Recruits (log N x 1e+6)
10
15
20
1970
1980
1990
2000
2010
Year
cfg_XI_op
cfg_I_est
cfg_V_est
cfg_X_est
cfg_XI_est
cfg_XVI_est

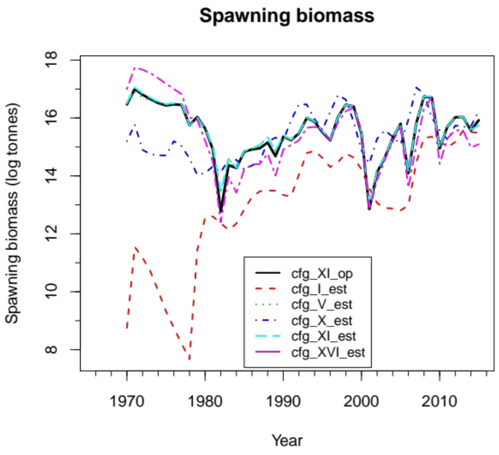
Spawning biomass
Spawning biomass (log tonnes)
8
10
12
14
16
18
1970
1980
1990
2000
2010
Year
cfg_XI_op
cfg_I_est
cfg_V_est
cfg_X_est
cfg_XI_est
cfg_XVI_est
